# Second Primary Malignancy after Acute Promyelocytic Leukemia: A Population-Based Study

**DOI:** 10.3390/cancers12123610

**Published:** 2020-12-03

**Authors:** Luana Lenzi, Lisa Lee-Jones, Maruf A. Mostofa, Diancarlos P. de Andrade, Raul C. Ribeiro, Bonald C. Figueiredo

**Affiliations:** 1 Departamento de Análises Clínicas, Universidade Federal do Paraná, Curitiba, Paraná 80210-170, Brazil; luanalnz@ufpr.br; 2 Life Sciences Department, Manchester Metropolitan University, Manchester M1 5GD, UK; l.lee-jones@mmu.ac.uk (L.L.-J.); MARUF.MOSTOFA@stu.mmu.ac.uk (M.A.M.); 3 Instituto de Pesquisa Pelé Pequeno Príncipe, Curitiba, Paraná 80250-060, Brazil; diancarlos.andrade@pelepequenoprincipe.org.br; 4 Pele Pequeno Principe Research Institute, Faculdades Pequeno Príncipe, Curitiba, Paraná 80230-020, Brazil; 5 Leukemia and Lymphoma Division, Department of Oncology, St. Jude Children’s Research Hospital, Memphis, TN 38105, USA; 6 Centro de Genética Molecular e Pesquisa do Câncer em Crianças (CEGEMPAC), Curitiba, Paraná 80030-110, Brazil; 7 Departamento de Saúde Coletiva, Universidade Federal do Paraná, Curitiba, Paraná 80060-240, Brazil

**Keywords:** leukemia, APL, acute promyelocytic, ATRA, chemotherapy

## Abstract

**Simple Summary:**

Acute promyelocytic leukemia (APL) is a rare and aggressive subtype of acute myeloid leukemia (AML). Since the introduction of all-trans-retinoic acid (ATRA) in APL management, the survival rate has increased substantially. However, there is evidence that retinoids might enhance tumor growth and the risk of secondary malignancies. The relationship between secondary cancer risk and APL treatment that includes ATRA is incompletely characterized. In this study, we investigated the risk factors associated with second primary malignancies after treatment of APL. Age ≥ 40 years at diagnosis of APL was significantly associated with an increased risk of second malignancies. Our findings suggest a potential carcinogenic role for ATRA in the salivary gland, liver, and soft tissue malignancies. Moreover, secondary tumors were significantly more frequent among patients with primary APL than in individuals with non-APL malignancies. Our finding suggests opportunities for surveillance for patients who completed treatment for APL.

**Abstract:**

Acute promyelocytic leukemia (APL), is now highly curable with treatment approaches that include all-trans retinoic acid (ATRA). The high incidence of APL in the Hispanics suggests an association with genetic variants in this population. Information on second primary malignancies (SPMs) in patients with APL is limited. The Surveillance, Epidemiology, and End Results (SEER) database was used to interrogate whether the rate of SPMs in patients with APL was associated with ethnicity and/or ATRA treatment. Between 2000 and 2016, 116 cases of SPM were diagnosed among 4019 patients with APL. The mean age at diagnosis of primary APL was 53.9 years (±15.7 years), and the mean age at diagnosis of SPMs was 59.0 years (±14.5 years). Comparisons with 3774 APL survivors who did not develop SPMs revealed that age ≥40 years at diagnosis of APL (*p* < 0.001) and non-Hispanic white ethnicity (*p* = 0.025) were associated with SPMs in APL survivors. Salivary gland, liver, and soft tissue malignancies were significantly more common in patients with primary APL than in individuals with non-APL malignancies. A risk analysis comparing patients who had APL with patients who had non-APL AML suggests that SPMs after APL is associated with ATRA treatment. Therefore, patient follow-up after APL should focus on early diagnosis of SPMs.

## 1. Introduction

Acute promyelocytic leukemia (APL) is a subtype of acute myeloid leukemia (AML) that is characterized by excessive proliferation of promyelocytes [1]. It is a highly aggressive hematopoietic neoplasm that is associated with rearrangements between the genes encoding retinoic acid receptor alpha (RARα) or other members of the retinoic acid receptor (RAR) family and several partner genes [2,3]. The most common rearrangement is a reciprocal translocation involving chromosomes 15 and 17 [t(15;17)(q24;q21)] that results in an abnormal fusion gene identified as Promyelocytic Leukemia/Retinoic Acid Receptor Alpha (PML/RARα) [4]. APL was once considered invariably fatal, but therapeutic advances, including administration of all-trans retinoic acid and arsenic trioxide (ATRA-ATO), have made it one of the most curable variants of AML, with more than 90% of patients experiencing complete remission [5,6] and more than 80% of them having 5-year disease-free survival [4,7]. 

The proportion of cases of APL relative to other AML subtypes is higher in Latin American countries (28.2% in Brazil; 27.8% in Venezuela; 22% in Peru; 20% in Mexico) than in European countries (10% in the UK and Scandinavia; 11.5% in Italy) [8]. The higher incidence in Latin America is yet to be explained but could be associated with environmental and/or ethnicity factors [8,9].

Since APL was first described in 1957 [10], its management has undergone many changes, evolving from antimetabolite-based therapy to anthracycline and cytarabine–based chemotherapy between 1980 and 1988 [11]. In 1985, ATRA was first used to manage APL. ATRA demonstrated clinical efficacy when used alone, substantially increasing the incidence of complete remission (CR) but not its durability [12,13]. However, a combination of ATRA with conventional AML chemotherapy was associated with improved outcomes and eradication of APL [11]. In the 1990s, ATO was introduced to treat relapsed APL, which resulted in higher CR rates [14]. Numerous studies demonstrated that ATRA-ATO combination therapy prolonged survival and eradicated the disease in low-risk patients with APL [11]; however, there have been no publications concerning second primary malignancies (SPMs) in patients treated with ATRA-containing regimens.

Cancer treatment in the form of chemotherapy and/or radiotherapy can cause the development of an SPM. Despite their increased survival rate, APL survivors can develop secondary malignancies [15]. In a 3-year follow-up study of patients with APL treated with ATRA-containing regimens, 8% of the patients developed new malignancies [16]. However, the incidence and type of SPMs in patients with APL is still poorly characterized [15]. Our study analyzed population-based data on SPMs in patients treated for APL. For comparison, we also analyzed data on SPMs in individuals with non-APL primary malignancies. We sought to determine whether the incidence of SPMs was increased among patients treated for APL and whether ethnicity and treatment had any impact on the rates of SPM in these patients. We compared the risk of SPMs in patients who had APL with that in patients who had non-APL cancers of any type and that in patients who had non-APL AML.

## 2. Results

### 2.1. Baseline Characteristics

**Figure 1:**
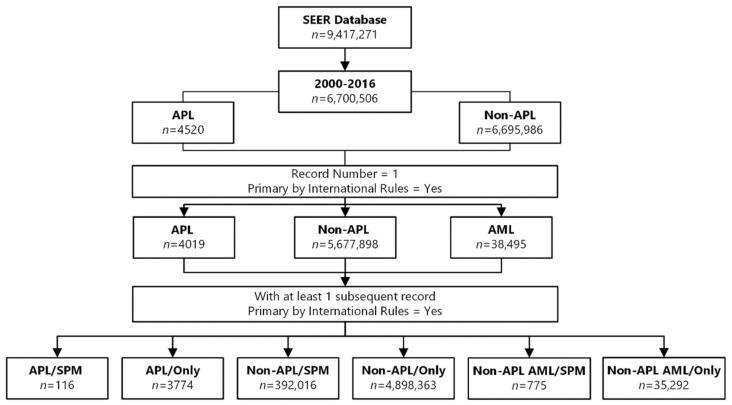
Flow chart showing the selection of groups from the Surveillance, Epidemiology, and End Results (SEER) 18 database (with additional treatment fields, November 2018 submission data) from 2000 to 2016.

Between 2000 and 2016, a total of 4520 patients with a diagnosis of APL were reported in the Surveillance, Epidemiology, and End Results (SEER) 18 registry. Of these patients, 4019 were classified as having a primary disease by the International Agency for Research on Cancer (IARC)/WHO [17] and their cancers were registered as first cancers (Figure 1). In 501 records, APL was not the first cancer and/or it was not classified as a primary disease; these patients were, therefore, excluded from the analysis. A record of SPM, according to the WHO/IARC rules, was identified for 116 patients (the APL/SPM group). The mean age at diagnosis of APL was 53.9 years (±15.7 years; median 56; interquartile range [IQR]: 46.5–65.0), and the mean age at diagnosis of the SPM was 59.0 years (±14.5 years; median 61; IQR: 54.0–70.0). Of these 116 patients, 60 (51.7%) were male and the median follow-up time for 114 of the patients was 7.8 years (range, 0.2–16.9 years). In two cases, both primary malignancies were diagnosed simultaneously, whereas the median latency period for SPM development of the other 114 patients was 4 years (range, 0.1–14.0 years). The APL/only group comprised 3774 patients with de novo APL who did not develop an SPM. In this group, 1919 patients (50.8%) were male, the mean age was 45.4 years (±19.3 years), and the median follow-up time was 4.8 years (range, 0.1–16.9 years).

The non-APL group represented all records of cancers other than APL (*n* = 6,695,986), of which 1,018,088 records were of non-APL cancers (including non-APL AML) that were not first cancers and/or were not classified as primary malignancies and were, therefore, excluded from the analysis. An SPM was observed in 392,016 cases, and 109,533 records were excluded because the second malignancy was not classified as a primary malignancy. Non-APL cancer was the only malignancy in 4,898,363 registry records, and 277,966 records were excluded because the sequence of the cancers was unknown. The mean age of this group was 66.3 years (±11.8) years), 236,522 (60.3%) of the patients were male, and the median follow-up time for 389,005 patients was 6.3 years (range, 0.1–16.9 years). In 3011 cases, both primary malignancies were diagnosed simultaneously. The mean age at diagnosis of the SPM was 70.1 years (±11.7 years), and the median latency period for SPM development was 3 years (range, 0–16.0 years). Finally, the non-APL group included 38,495 patients who had non-APL AML as their first malignancy. A second malignancy was observed in 933 records, 2270 records were excluded because the sequence of the cancers was unknown, and 35,292 patients had non-APL AML as their only cancer. The second malignancy was classified as primary in 775 patients, who were selected as the non-APL AML group. In the non-APL AML group, the mean age at diagnosis was 61.2 years (±16.6 years), 464 (59.9%) of the patients were male, and the median follow-up time for 727 patients was 2.9 years (0.1–16.7 years). In 48 cases, both primary malignancies were diagnosed simultaneously. The mean age at SPM diagnosis was 63.8 years (±15.7 years), and the median latency period was 1.0 years (range, 0–16 years). The characteristics of all of the study groups are summarized in Table 1.

Comparing the baseline characteristics of the APL/SPM, APL/only, non-APL, and non-APL AML groups (Table 2), it is apparent that the frequency of SPMs was higher in the non-APL group (*p* < 0.001) than in the other groups and equivalent in the non-APL AML and APL groups (*p* = 0.193). With regard to age, the two groups of patients with APL (the APL/only and APL/SPM groups) contained more patients younger than 40 years when compared to the non-APL groups; however, the proportion of patients older than 40 years was higher among patients with APL who had developed an SPM (*p* < 0.001). The proportion of Hispanic patients was higher in the APL groups (12.1% in the APL/SPM group and 24.1% in the APL/only group), whereas the proportion of non-Hispanic (white) patients was higher in the non-APL group (77.7%) (*p* < 0.001). Comparisons between the APL/SPM and non-APL AML/SPM groups showed that the proportion of non-Hispanic white patients was 10.7% higher in the non-APL AML/SPM group and that the proportion of Hispanic patients was 3.3% smaller in that group (*p* < 0.001). 

To determine whether the risk of SPM for patients with APL was higher than that for individuals with other cancer types, we compared the risk of SPM for the APL, non-APL, and non-APL AML groups. Groups were combined to determine the risk associated with the development of an SPM after APL (APL/SPM vs. APL/only); which cancer types were most likely to develop after APL (APL/SPM vs. non-APL/SPM); and whether chemotherapy was related to the SPM (APL/SPM vs. non-APL AML/SPM).

### 2.2. Second Primary Malignancies

The frequency of SPMs differs between patients with APL and non-APL patients. In the APL/SPM cohort, 2.9% of survivors developed an SPM whereas the frequency in the non-APL group was 6.9% (*p* < 0.001) and the frequency in the non-APL AML group was 2.0% (*p* = 0.193). To investigate the factors associated with SPM development after APL, a time-dependent Cox regression model was applied to data from the APL/SPM and APL/only groups (Table 3). The variables analyzed included sex, age, and race/ethnicity, controlling for the follow-up time. According to the results, patients aged at least 40 years at diagnosis of APL had a 5.1-fold increased risk of developing an SPM when compared with those younger than 40 years (*p* < 0.001). All non-Hispanic groups had a higher risk of developing an SPM when compared to Hispanic patients.

**Figure 2:**
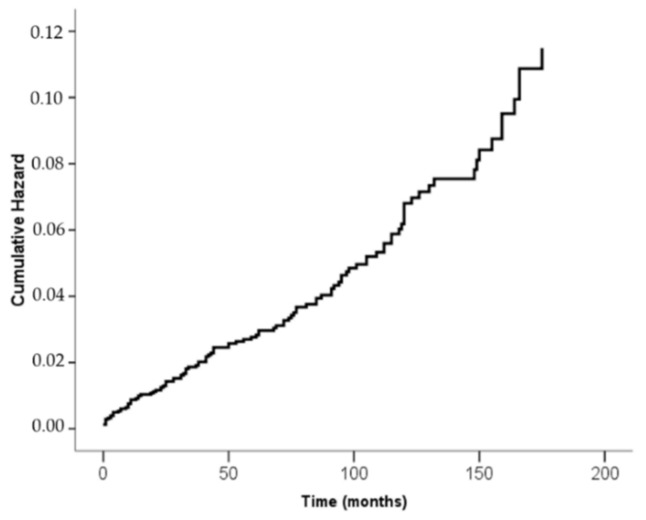
Cumulative incidence of SPMs after APL represented by the hazard ratio with the latency time for the development of the SPM adjusted by the follow-up time.

The cumulative incidence of SPM after APL is illustrated in Figure 2. The latency time for the occurrence of the SPM was adjusted by the follow-up time and indicates that after 10 years the risk of developing an SPM is around 7%. 

**Figure 3:**
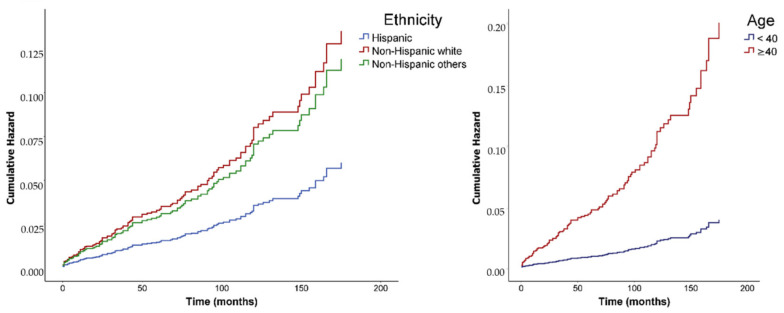
Hazard ratios for second primary malignancy after APL, comparing ethnicity and age at diagnosis of APL.

As illustrated in Figure 3, the risk of developing an SPM after APL increases with time. At 10 years after APL, the risk of non-Hispanic APL survivors developing an SPM is approximately three times that in Hispanic survivors (*p* = 0.016). Increasing risk over time is also observed in APL survivors when they are compared by age. When APL is diagnosed after 40 years of age, the risk of having an SPM after 10 years is around four times higher than for those survivors in whom APL is diagnosed at a younger age (*p* < 0.001).

**Figure 4:**
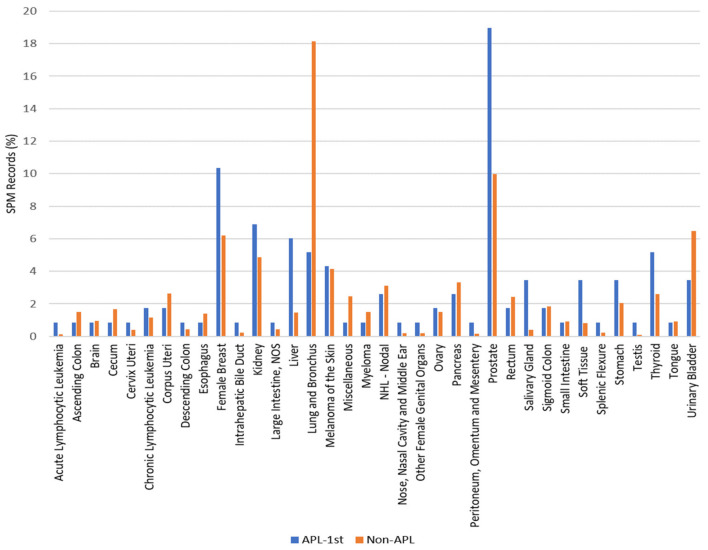
Sites of second primary malignancies in APL survivors and non-APL survivors between 2000 and 2016.

As illustrated in Figure 4, the frequency of cancer sites differed significantly between the APL/SPM and non-APL/SPM groups. Cancers were more frequent in the female breast (*p* = 0.0009), kidney (*p* = 0.0454), liver (*p* < 0.001), prostate (*p* < 0.001), salivary gland (*p* < 0.001), soft tissue (*p* < 0.001), and thyroid (*p* = 0.0027) in the APL/SPM group, whereas cancers of the lung and bronchus (*p* < 0.001) and bladder (*p* = 0.0014) were more frequent in the non-APL group.

To identify the cancers that had a higher risk of occurrence as an SPM after APL, the APL/SPM group was compared with the non-APL/SPM group. A Poisson regression model was used to assess the relative risk (RR) and CI95% (Table 4). An overall analysis including all patients showed that patients with APL/SPM had an increased risk of developing cancers of the liver (RR: 4.6; *p <* 0.001), salivary gland (RR: 9.8; *p* < 0.001), and soft tissue (RR: 4.7; *p* = 0.003) when compared with non-APL patients. Furthermore, the risks varied according to sex. Men who had APL as first cancer had additional risks of 3.3, 4.0, and 2.5 for cancer of the liver, salivary gland, and soft tissue, respectively, when compared to women. The risk of prostate cancer after primary APL was 3.7 times the risk after non-APL primary malignancies. However, a comparison of the APL/SPM and non-APL groups showed no differences in the risk of women developing breast cancer (*p* = 0.057).

Incidences rates for liver, salivary gland and soft tissue cancer were compared between APL survivors and the general population (identified by all cancer registries from SEER 18 classified as primary by international rules). Table 5 shows that all absolute incidences rates for the identified cancers were higher in individuals after treatment for APL.

The time to develop SPMs varied significantly between APL/SPM survivors (median, 4 years; range, 0–14 years) and non-APL survivors (median, 3 years; range, 0–16 years) (*p* = 0.001). By using the multiple primary–standardized incidence ratios (MP-SIR) session of SEER*Stat, we assessed the incidence of SPMs after APL and compared it with the incidence of the same tumors in the non-APL patients and the general population. In this way, we identified the cancers with a higher incidence ratio after APL (Table 6).

Considering the overall survival time, the only cancers with a significantly higher risk of SPM development after APL were cancers of the salivary gland, liver, and soft tissue, which was consistent with our initial findings with the Poisson regression model.

### 2.3. Association of SPMs with the Therapy of Myeloid Neoplasms

Because the chemotherapy regimens for APL and non-APL AML differed essentially with respect to the inclusion of ATRA during the study period (2000 to 2016), we compared the SPM records for each group (APL/SPM vs. non-APL AML/SPM) to assess the RR. As shown in Table 7, cancers of the salivary gland, liver, soft tissue, and prostate had a higher risk of giving rise to an SPM after APL than after a non-APL AML. There was no significant difference between the risk of soft tissue cancer in the APL/SPM group and that in the non-APL AML group. 

## 3. Discussion

Our study revealed that the overall rates of SPMs after treatment of APL were significantly lower than those in individuals treated for non-APL malignancies (*p* < 0.001). These findings were expected, given that non-APL malignancies include various subtypes, many of which are treated with radiotherapy and/or chemotherapy agents (alkylators, topoisomerase II inhibitors, platinum), which predispose the recipients to second cancers [18]. Although the incidence of SPMs was comparable among patients with APL or non-APL AML (*p* = 0.193), cancer profiling differed between these two groups. Recognizing the impact of anticancer treatment on the development of second neoplasms, we chose to limit our study to the period between 2000 and 2016 because the combination of ATRA, anthracycline, and cytarabine was commonly used in the management of APL during that period [11]. Moreover, the diagnosis of second malignancies in our study followed strict criteria recommended by IARC/WHO [17]. Several studies have investigated the risks of the second malignancies in patients with APL [15,19,20,21]. However, these studies differed from ours in several aspects, including treatment heterogeneity, the age of the patients at diagnosis, and second malignancy ascertainment criteria. These factors might explain the differences in the risk rates for secondary malignancies in the APL studies.

Remarkably, the risk rates for SPMs varied with age at diagnosis of APL. Our analysis showed that patients who were at least 40 years of age when their APL was diagnosed had a 5.1-fold higher risk of developing an SPM when compared with patients who were younger than 40 years when their APL was diagnosed. The observation that age at diagnosis of APL was associated with the risk of SPMs irrespective of the follow-up time and latency period was unexpected. More commonly, secondary cancers that develop after treatment occur after a latency period of several months to several years, depending on the therapy used but not on the age of the patient at diagnosis of the primary tumor. For example, children and adults treated with chemotherapy regimens that include topoisomerase II inhibitors have an increased risk of developing AML, typically within 3 years after the exposure [18].

In contrast, patients with tumors that were managed with alkylating agents and/or radiotherapy had an increased risk of developing neoplasms, usually 10 or more years after the exposure [18,22]. The association of SPMs with older age suggests that the chemotherapy used for APL interacts with ageing-associated changes to induce tumor formation. Similar to the observation in studies of clonal hematopoiesis of indeterminate potential [23], age-associated somatic mutations occur naturally in several organs and tissues, including the skin, salivary glands, thyroid, muscle, breast, and prostate, among others [22]. Chemotherapy combinations used to treat APL, including a combination of anthracycline, cytarabine, and ATRA might exert selective pressure in certain tissues, accelerating clonal evolution and leading to secondary tumors. Whether the introduction of ATO and decrease in conventional chemotherapy will affect SPM incidence and profiling in newly diagnosed APL remains elusive.

Our study corroborates earlier findings that the incidence of APL is higher in Hispanic populations than in non-Hispanic white populations [24]. There is a marked disproportion in the occurrence of APL in Hispanic and non-Hispanic white populations at younger ages [25]. Furthermore, Hispanic populations have a higher rate of acute lymphoblastic leukemia, typically in children and adolescents [18]. Genome-wide association studies have revealed that germline genetic variations, including variations in ARID5B, GATA3, PIP4K2A, and ERG, are common in Hispanic populations, and this partly explains the excess of acute lymphocytic leukemia (ALL) cases among Hispanic children and adolescents [26]. Because of the early age of onset of APL and the high rate of ALL in Hispanic populations, we reasoned that Hispanic patients treated for APL would have a significantly higher incidence of SPMs when compared with non-Hispanic white patients. However, our findings did not substantiate this hypothesis: non-Hispanic white patients had a significantly higher rate of SPMs when compared to Hispanic patients (HR: 2.3; CI95%: 1.3–4.0; *p* = 0.005). 

Because the treatment of APL exposes patients to ATRA during the induction, consolidation, and maintenance phases, we compared the APL profile of the patients with SPMs with those of patients with non-APL neoplasms or AML. The RR of developing neoplasms of the liver, salivary gland, or soft tissue was significantly higher for patients who had been treated for APL than for patients who had been treated for non-APL neoplasms or AML. When the RR of cancers was analyzed by sex, the risk of prostate cancer was also significantly higher for patients treated for APL than for patients treated for non-APL neoplasms or AML. These observations suggest that ATRA might contribute to the SPM profile observed among APL patients. 

In general, the physiologic retinoid activity requires the biotransformation of retinol to ATRA by the retinol dehydrogenase and reductase enzymes. ATRA binds to cellular retinoic acid-binding proteins and nuclear RARs. The RARs heterodimerize with retinoid X receptors to regulate the transcription of at least 500 genes [27]. At physiologic levels, retinoids are considered to have cancer-protective effects; however, at supraphysiologic levels, ATRA could promote tumorigenesis [28]. Using a murine sarcoma model, investigators found that cells from the tumor microenvironment induce tumor cells to produce retinoic acid, which stimulates tumor-associated macrophages, resulting in an immunosuppressive environment and tumor promotion [29]. The results of this study demonstrating a link between tumorigenesis and retinoic acid signaling might explain, in part, the propensity for secondary tumors to develop in patients in whom APL is diagnosed at an older age. 

The limitations of this study include the absence of detailed information such as symptoms, data on minimal residual disease and karyotype, specific drugs and dosages used for treatment, and stem cell transplantation in the SEER database preventing the execution of more specific analyzes.

## 4. Materials and Methods 

### 4.1. Data Source

The Surveillance, Epidemiology, and End Results (SEER) Program database (SEER*Stat version 8.3.6) was used in this study to conduct a retrospective analysis. APL cases were selected using ICD-O-3 code 9866 (Acute promyelocytic leukemia (AML with t(15;17)(q22;q12)) PML/RARA in the SEER 18 registry with additional treatment fields (November 2018 submission data). Data including age, sex, race/ethnicity, year of diagnosis, type of cancer, therapy, number of malignancies, and primary classification by international rules (i.e., according to the International Agency for Research on Cancer/World Health Organization (IARC/WHO) multiple primary rules) [17] were retrieved. Information regarding symptoms, minimal residual disease, drugs, cytogenetics, and stem cell transplantation status were unavailable. 

### 4.2. Study Population

The SEER database includes cancer cases diagnosed between 1975 and 2016, and the interval between 2000 and 2016 was selected for this study. The case listing option was used, and the person selection option specifying registers with at least one subsequent record was selected. To address the question regarding SPMs, three groups were defined. The APL/SPM group corresponded to patients who developed APL (ICD-O-3 code 9866) as their first cancer, which was classified as primary cancer by international rules, and who subsequently developed at least one additional cancer. Using filters, all cases were selected in which a second malignancy was also classified as primary cancer by international rules. The non-APL group corresponded to patients who had first developed any type of cancer other than APL before developing an SPM. The non-APL AML group comprised patients who had a subtype of AML that was not APL as their first primary malignancy and subsequently developed an SPM. Finally, the APL/only, non-APL/only, and non-APL AML/only groups represented those patients who had only the cancer of interest in each group (classified as primary by international rules, sequence number identified as one primary only) with no registry record of subsequent cancer. 

### 4.3. Ethics

A SEER Research Data Agreement was obtained to enable access to the SEER data. This study required no additional ethical approval as it involved no interaction with human participants or personal identification of participants. Therefore, informed consent was also unnecessary. 

### 4.4. Statistical Methods

Baseline characteristics were addressed using descriptive statistics, including absolute and relative frequencies. The chi-square test was used for comparisons of categorical variables and the Mann–Whitney U test for comparisons of continuous variables. The analyses were performed using the IBM SPSS (Statistical Package for the Social Sciences) software version 17.0. The latency time was determined using the date of diagnosis of the first cancer and the date of diagnosis of the second primary malignancy or the end of follow-up. The follow-up time was calculated using the date of diagnosis of the first cancer and the end of follow-up or death due to the SPM. To assess the factors influencing the occurrence of an SPM in patients with a prior primary APL, a time-dependent Cox regression model was applied to the APL/SPM group, using the APL/only group as the comparator. The model was adjusted with respect to the follow-up time, and the results were obtained using hazard ratios (HRs) with a 95% confidence interval (CI95%). 

To assess the sites with a higher risk of cancer development after APL, a Poisson regression analysis was conducted which compared the APL/SPM and non-APL groups, and the results were evaluated with respect to relative risk (RR) and CI95%. The multiple primary–standardized incidence ratios (MP-SIR) session of SEER*Stat software version 8.3.6 was used to explore theoretical links between the etiologies of the primary and secondary cancers. Two defined cohorts previously diagnosed with APL and non-APL cancer, respectively, were followed over time, comparing their subsequent cancer incidence to the number of cancers that would be expected based on the incidence rates for the general population. Excess risk per 10,000 individuals was calculated and a CI95% was obtained. Only results with statistical significance (*p* < 0.05) were shown. To evaluate the association of ATRA with SPM after APL, the APL/SPM and non-APL AML groups (which received similar chemotherapy differing only in the use of ATRA for the patients with APL) were used to compare the risks of developing an SPM at each potential cancer site; the results were presented as RR and CI95% values. 

## 5. Conclusions

In conclusion, our study has revealed that age of more than 40 years at diagnosis of APL, but not the follow-up time or the latency period, is associated with the risk of developing SPM after treatment with regimens that include anthracycline plus ATRA. The risk of developing an SPM of the salivary gland, liver, prostate, or soft tissue is significantly higher in survivors treated for APL than in patients treated for non-APL malignancies or AML. Surveillance, screening, and education of APL survivors who received ATRA-containing regimens should, therefore, focus on early detection of the most common SPMs and on the avoidance of factors typically associated with an increased risk of oral and liver malignancies.

## Figures and Tables

**Table 1:** Baseline characteristics of the different cohorts.

Variables			APL	Non-APL	Non-APL AML
Total number of records			4520	6,695,986	51,501
First cancer and primary by WHO rules			4019	5,677,898	38,495
First primary malignancy only			3774	4,898,363	35,292
	Mean age at diagnosis in years (SD)		45.4 (19.3)	63.1 (15.7)	61.1 (21.9)
	Sex				
		Female	1.855 (49.2)	2,395,182 (48.9)	16,145 (45.7)
		Male	1.919 (50.8)	2,503,181 (51.1)	19,147 (54.3)
	Ethnicity				
		Hispanic (all races)	908 (24.1)	511,927 (10.5)	4475 (12.7)
		Non-Hispanic (white)	766 (20.3)	3,451,992 (70.5)	24,540 (69.5)
		Non-Hispanic (other)	2100 (55.6)	934,444 (19.0)	6277 (17.8)
	Median follow-up time in years (range)		4.8 (0.1–16.9)	3.1 (0.1–16.9)	0.5 (0.1–16.9)
Second primary malignancy			116	392,016	755
	Mean age at diagnosis of the first malignancy in years (SD)		53.9 (15.7)	66.3 (11.8)	61.2 (16.6)
	Mean age at diagnosis of the SPM in years (SD)		59.0 (14.5)	70.1 (11.7)	63.8 (15.7)
	Sex				
		Female	56 (48.3)	155,494 (39.7)	311 (40.1)
		Male	60 (51.7)	236,522 (60.3)	464 (59.9)
	Ethnicity				
		Hispanic (all races)	14 (12.1)	27,787 (7.1)	68 (8.8)
		Non-Hispanic (white)	78 (67.2)	304,360 (77.7)	604 (77.9)
		* Non-Hispanic (other)	24 (20.7)	59,869 (15.2)	103 (13.3)
	Median latency time in years (range)		4 (0.1–14)	3 (0–16)	1 (0–16)
	Median follow-up time in years (range)		7.8 (0.2–16.9)	6.3 (0.1–16.9)	2.9 (0.1–16.7)

* Blacks, Asian or Pacific Islander, American Indian or Alaska Native. Abbreviations: AML, acute myeloid leukemia; APL, acute promyelocytic leukemia; SD, standard deviation; SPM, second primary malignancy; WHO, World Health Organization.

**Table 2:** Comparisons of baseline characteristics.

Baseline Characteristics		APL/SPM	APL/only	*p* *	Non-APL/SPM	*p* *	Non-APL AML/SPM	*p* ^#^
Frequency of SPM		2.9%	---	---	6.9%	<0.001	2.0%	0.193
Age								
	<40 years	18 (15.5)	1486 (39.4)	<0.001	7667 (2.0)	<0.001	70 (9.0)	<0.001
	≥40 years	98 (84.5)	2288 (60.6)		384,349 (98.0)		705 (91.0)	
Sex								
	Female	56 (48.3)	1.855 (49.2)	0.942	155,494 (39.7)	0.058	311 (40.1)	0.096
	Male	60 (51.7)	1.919 (50.8)		236,522 (60.3)		464 (59.9)	
Ethnicity								
	Hispanic	14 (12.1)	908 (24.1)	0.001	27,787 (7.1)	<0.001	68 (8.8)	<0.001
	Non-Hispanic (white)	78 (67.2)	2100 (55.6)		304,360 (77.7)		604 (77.9)	
	* Non-Hispanic (other)	24 (20.7)	766 (20.3)		59,869 (15.2)		103 (13.3)	
Median follow-up time in years (range)		7.8 (0.2–16.9)	4.8 (0.1–16.9)	<0.001	6.3 (0.1–16.9)	0.001	2.9 (0.1–16.7)	<0.001

* Blacks, Asian or Pacific Islander, American Indian or Alaska Native. ^#^ z-test. Abbreviations: AML, acute myeloid leukemia; APL, acute promyelocytic leukemia; SPM, second primary malignancy; WHO, World Health Organization.

**Table 3:** Factors associated with the development of a second primary malignancy after APL, comparing the APL/only and APL/SPM groups by using a time-dependent Cox regression model.

Risk Factors		APL/only *n* (%)	APL/SPM *n* (%)	HR (CI95%)	*p* ^#^
Age					
	<40 years	18 (15.5)	1486 (39.4)	---	
	≥40 years	98 (84.5)	2288 (60.6)	5.1 (3.1–8.4)	<0.001
Race/ethnicity					
	Hispanic (all races)	14 (12.1)	908 (24.1)	---	0.020
	Non-Hispanic white	78 (67.2)	2100 (55.6)	2.3 (1.3–4.0)	0.005
	* Non-Hispanic (other)	24 (20.7)	766 (20.3)	2.0 (1.0–3.8)	0.041

* Black, Asian or Pacific Islander, American Indian or Alaska Native. ^#^ Time-dependent Cox regression model, Wald test. Abbreviations: APL, acute promyelocytic leukemia; HR, hazard ratio; CI, confidence intervals; SPM, second primary malignancy.

**Table 4:** Sites with a high risk of cancer occurrence after APL, evaluated as relative risk obtained by a Poisson regression model for the period 2000–2016, overall and according to sex.

Second Primary Malignancy	APL/SPM *n* (%)	Non-APL/SPM *n* (%)	RR (CI95%)	*p* *
Overall	116	392,016		
Liver	7 (6.0)	5719 (1.5)	4.6 (2.1–9.9)	<0.001
Salivary gland	4 (3.4)	1542 (0.4)	9.8 (3.6–26.5)	<0.001
Soft tissue	4 (3.4)	3241 (0.8)	4. 7 (1.7–12.6)	0.003
In men	60	236,522		
Liver	5 (8.3)	4325 (1.8)	7.6 (2.9–19.6)	<0.001
Salivary gland	2 (3.3)	937 (0.4)	14.0 (3.3–58.6)	<0.001
Soft tissue, including heart	2 (3.3)	1911 (0.8)	6.9 (1.6–28.7)	0.008
Prostate	22 (36.7)	39,068 (16.5)	3.7 (2.1–6.4)	<0.001
In women	56	155,494		
Liver	2 (3.6)	1394 (0.9)	4.4 (1.1–17.9)	0.041
Salivary gland	2 (3.6)	605 (0.4)	10.0 (2.4–41.2)	0.001
Soft tissue	2 (3.6)	1330 (0.9)	4.571 (1.1–18.8)	0.035
Female breast	12 (21.4)	24,270 (15.6)	2. 5 (0.9–6.3)	0.057

* Poisson regression, Wald chi-square. Abbreviations: APL, acute promyelocytic leukemia; CI, confidence interval; RR, relative risk; SPM, second primary malignancy.

**Table 5:** Incidence rates per 100,000 for liver, salivary gland and soft tissue cancer classified as primary, compared between all registries of SEER 18 and APL survivors for the period 2000–2016, according to sex and age.

Cancer		Incidence/100.000 (2000–2016)		
		All Registries SEER 18 *	All APL *	*p*
Liver				
Sex	Female	3.8	6.2	0.016
	Male	10.9	14.9	0.013
Age	<40	0.3	0.0	0.317
	≥40	16.1	17.3	0.511
Salivary Gland				
Sex	Female	1.1	6.2	<0.001
	Male	1.5	8.9	<0.001
Age	<40	0.3	7.8	<0.001
	≥40	2.6	7.4	<0.001
Soft Tissue				
Sex	Female	2.9	9.2	<0.001
	Male	3.7	5.9	0.025
Age	<40	1.4	7.8	<0.001
	≥40	5.7	7.4	0.137

* All primary malignancies. Abbreviations: APL, acute promyelocytic leukemia; SEER, surveillance epidemiology and end results program.

**Table 6:** Overall significant standardized incidence ratio (SIR) of second primary malignancy after APL and in non-APL survivors.

Cancer Sites in SPM		Observed	Expected	SIR	CI95%	Excess Risk
APL/SPM						
	Salivary gland	4	0.31	12.89	(3.51–33.01)	2.02
	Liver	6	2.07	2.90	(1.07–6.32)	2.16
	Soft tissue	4	0.77	5.18	(1.41–13.25)	1.77
Non-APL/SPM						
	Salivary gland	1775	1191.23	1.49	(1.42–1.56)	0.2
	Liver	6254	6710.05	0.93	(0.91–0.96)	−0.16
	Soft tissue	4025	2440.49	1.65	(1.6–1.7)	0.56

Abbreviations: APL, acute promyelocytic leukemia; SPM, second primary malignancy; SIR, standardized incidence ratio; CI, confidence interval.

**Table 7:** Association of second primary malignancies after APL with ATRA treatment by comparing APL/SPM and non-APL AML groups.

Second Primary Malignancy Site	APL/SPM (*n* = 116) *n* (%)	Non-APL AML (*n* = 775) *n* (%)	RR (CI95%)	*p* *
Overall				
Liver	7 (6.0)	4 (0.5)	5.437 (2.528–11.697)	<0.001
Salivary gland	4 (3.4)	3 (0.4)	4.883 (1.798–13.262)	0.002
Soft tissue	4 (3.4)	6 (0.8)	3.418 (1.258–9.283)	0.016
In men	60	464		
Liver	5 (8.3)	4 (0.9)	7.682 (2.974–19.845)	<0.001
Salivary gland	2 (3.3)	2 (0.4)	6.914 (1.650–28.974)	0.008
Soft tissue	2 (3.3)	3 (0.6)	5.531 (1.320–23.180)	0.019
Prostate	22 (36.7)	83 (17.9)	2.897 (1.665–5.042)	<0.001
In women	56	311		
Liver	2 (3.6)	0 (0.0)	7.140 (1.737–29.343)	0.006
Salivary gland	2 (3.6)	1 (0.32)	4.760 (1.158–19.562)	0.030
Soft tissue	2 (3.6)	3 (1.0)	2.856 (0.695–11.737)	0.146

* Poisson regression, Wald Chi-square. Abbreviations: AML, acute myeloid leukemia; APL, acute promyelocytic leukemia; ATRA, all-trans-retinoic acid; SPM, second primary malignancy; RR, relative risk; CI, confidence interval.
